# SUMO Modification of Histone Demethylase KDM4A in Kaposi’s Sarcoma-Associated Herpesvirus-Induced Primary Effusion Lymphoma

**DOI:** 10.1128/jvi.00755-22

**Published:** 2022-08-01

**Authors:** Wayne W. Yeh, Yin-Quan Chen, Wan-Shan Yang, Yung-Chih Hong, Sen Kao, Tze-Tze Liu, Ting-Wen Chen, Lung Chang, Pei-Ching Chang

**Affiliations:** a Institute of Microbiology and Immunology, National Yang Ming Chiao Tung University, Hsinchu, Taiwan; b Institute of Microbiology and Immunology, National Yang-Ming University, Taipei, Taiwan; c Cancer Progression Research Center, National Yang Ming Chiao Tung University, Taipei, Taiwan; d Faculty of Medicine, National Yang-Ming Chiao Tung University, Taipei, Taiwan; e Institute of Bioinformatics and Systems Biology, National Yang Ming Chiao Tung University, Hsinchu, Taiwan; f Center for Intelligent Drug Systems and Smart Bio-devices (IDS^2^B), National Yang Ming Chiao Tung University, Hsinchu, Taiwan; g Department of Pediatrics, MacKay Children’s Hospital and MacKay Memorial Hospital, Taipei, Taiwan; h Department of Medicine, MacKay Medical College, New Taipei, Taiwan; Lerner Research Institute, Cleveland Clinic

**Keywords:** KDM4A, Kaposi's sarcoma-associated herpesvirus (KSHV), SUMOylation, histone lysine demethylase (KDM), primary effusion lymphoma (PEL)

## Abstract

Primary effusion lymphoma (PEL) is a fatal B-cell lymphoma caused by Kaposi’s sarcoma-associated herpesvirus (KSHV) infection. Inducing KSHV lytic replication that causes the death of host cells is an attractive treatment approach for PE; however, combination therapy inhibiting viral production is frequently needed to improve its outcomes. We have previously shown that the KSHV lytic protein K-bZIP can SUMOylate histone lysine demethylase 4A (KDM4A) at lysine 471 (K471) and this SUMOylation is required for virus production upon KSHV reactivation. Here, we demonstrate that SUMOylation of KDM4A orchestrates PEL cell survival, a major challenge for the success of PEL treatment; and cell movement and angiogenesis, the cell functions contributing to PEL cell extravasation and dissemination. Furthermore, integrated ChIP-seq and RNA-seq analyses identified interleukin-10 (IL-10), an immunosuppressive cytokine, as a novel downstream target of KDM4A. We demonstrate that PEL-induced angiogenesis is dependent on IL-10. More importantly, single-cell RNA sequencing (scRNA-seq) analysis demonstrated that, at the late stage of KSHV reactivation, KDM4A determines the fates of PEL cells, as evidenced by two distinct cell populations; one with less apoptotic signaling expresses high levels of viral genes and the other is exactly opposite, while KDM4A-K417R-expressing cells contain only the apoptotic population with less viral gene expression. Consistently, KDM4A knockout significantly reduced cell viability and virus production in KSHV-reactivated PEL cells. Since inhibiting PEL extravasation and eradicating KSHV-infected PEL cells without increasing viral load provide a strong rationale for treating PEL, this study indicates targeting KDM4A as a promising therapeutic option for treating PEL.

**IMPORTANCE** PEL is an aggressive and untreatable B-cell lymphoma caused by KSHV infection. Therefore, new therapeutic approaches for PEL need to be investigated. Since simultaneous induction of KSHV reactivation and apoptosis can directly kill PEL cells, they have been applied in the treatment of this hematologic malignancy and have made progress. Epigenetic therapy with histone deacetylase (HDAC) inhibitors has been proved to treat PEL. However, the antitumor efficacies of HDAC inhibitors are modest and new approaches are needed. Following our previous report showing that the histone lysine demethylase KDM4A and its SUMOylation are required for lytic reactivation of KSHV in PEL cells, we further investigated its cellular function. Here, we found that SUMOylation of KDM4A is required for the survival, movement, and angiogenesis of lytic KSHV-infected PEL cells. Together with our previous finding showing the importance of KDM4A SUMOylation in viral production, KDM4A can be a potential therapeutic target for PEL.

## INTRODUCTION

Primary effusion lymphoma (PEL), characterized by a pleural or peritoneal lymphomatous effusion without solid tumor mass, is therefore also known as body cavity-based lymphoma (BCBL) (1). It is an AIDS-associated non-Hodgkin’s B-cell lymphoma caused by Kaposi’s sarcoma-associated herpesvirus (KSHV) infection (2) with a median survival of less than 2 years (3, 4). Since combined antiretroviral therapy (cART) enhanced the life span of AIDS patients but did not decrease the occurrence of AIDS-related lymphomas (ARLs) in the same extent (5, 6), ARL cases are expected to increase. Although the widely used standard chemotherapeutic regimen of cyclophosphamide, doxorubicin, vincristine, and prednisone (CHOP) has temporarily controlled the disease, the clinical outcome of PEL remains extremely poor and continues to cause death. Most patients experience relapse after successful treatment within 6 to 8 months (7, 8) and have a median overall survival of around 4.8 months (9). All this highlights the need for new therapeutic modalities for this malignancy.

The human oncogenic gammaherpesvirus KSHV, also known as human herpesvirus-8 (HHV-8), is an oncogenic DNA virus exhibiting biphasic life cycle: latency and lytic replication (10). Therapeutic induction of lytic replication of KSHV has been recognized as a promising approach to treat KSHV-associated hematological malignancies that cannot be surgically removed (11). During the latent state, the KSHV genome circularizes into a double-stranded episome, chromatinizes, and recruits cellular epigenetic regulators to form a repressive chromatin structure that restricts viral gene expression. Epigenetic changes can switch viral latency to lytic replication (12–15), and therefore histone deacetylase (HDAC) inhibitors have been used to induce KSHV reactivation (16) and have been considered a potential anticancer therapeutic option to treat PEL (17, 18). However, reactivation of herpesviruses will lead to robust virion production. Moreover, to ensure virion production, KSHV exploits multiple mechanisms to prevent apoptosis during lytic viral replication. These include expressing viral lytic antiapoptotic genes of vIAP (ORFK7) (19) and vBcl-2 (ORF16) (20) that inhibit mitochondrial apoptosis and of K-Rta (21), K-bZIP (22), ORF45 (23) that inhibit p53-induced apoptotic signaling. Therefore, concomitant inhibition of viral production and antiapoptotic signaling has been suggested to improve the therapeutic efficacy of HDAC inhibitors (17, 24). Understanding more about epigenetic regulation of KSHV reactivation will help develop epigenetic-targeted therapy to treat PEL successfully.

In particular, our previous report showed that the deposition of histone lysine demethylase 4A (KDM4A) on the KSHV episome limits the enrichment of heterochromatin mark H3K9me3 on certain parts of the viral genome that contain immediate early (IE) and early (E) genes, allowing rapid switch-on of the viral lytic cycle (25, 26). Knockdown of KDM4A significantly reduced lytic gene expression and KSHV production after reactivation (27). More importantly, we identified a SUMO modification site on KDM4A at the lysine (K)-471 residue and showed that SUMOylation is required for the chromatin binding and demethylase activity of KDM4A (25). Therefore, the SUMO-deficient mutant of KDM4A (KDM4A-K471R) cannot rescue the virus lytic gene expression and virion production in KDM4A knockdown BCBL-1 cells (25). Together with the long-acknowledged oncogenic role of KDM4A in inhibiting apoptosis (28, 29), we hypothesize that targeting KDM4A or its SUMO modification may reduce infectious viral particle production, prevent viral antiapoptotic effects, and be a potential therapeutic option to kill PEL cells.

To study this, we first performed Gene Ontology (GO) analysis using differentially expressed genes (DEGs) obtained from KDM4A-WT and KDM4A-K471R upon KSHV reactivation to determine the cellular mechanism by which KDM4A SUMOylation regulates during KSHV reactivation. We found that in addition to inhibiting cell apoptosis, KDM4A also promotes cell movement and angiogenesis, the cellular functions contributing to lymphoma dissemination (30, 31). Using single-cell RNA sequencing (scRNA-seq), we demonstrated that KDM4A protects a population of PEL cells undergoing late phase of KSHV reactivation from death. In addition, integrated ChIP-seq and RNA-seq analyses identified immunosuppressive cytokine IL-10 as a direct target of KDM4A and participated in PEL-mediated endothelial angiogenesis. Finally, using CRISPR/Cas9 knockout of KDM4A (KDM4A^KO^), we confirmed that loss of KDM4A could induce cell death, with minimal virion production, in KSHV-reactivated PEL cells. These results indicate that KDM4A would be a new promising therapeutic target for treating PEL.

## RESULTS

### Identification of cellular function regulated by KDM4A SUMOylation during KSHV reactivation.

**FIG 1:**
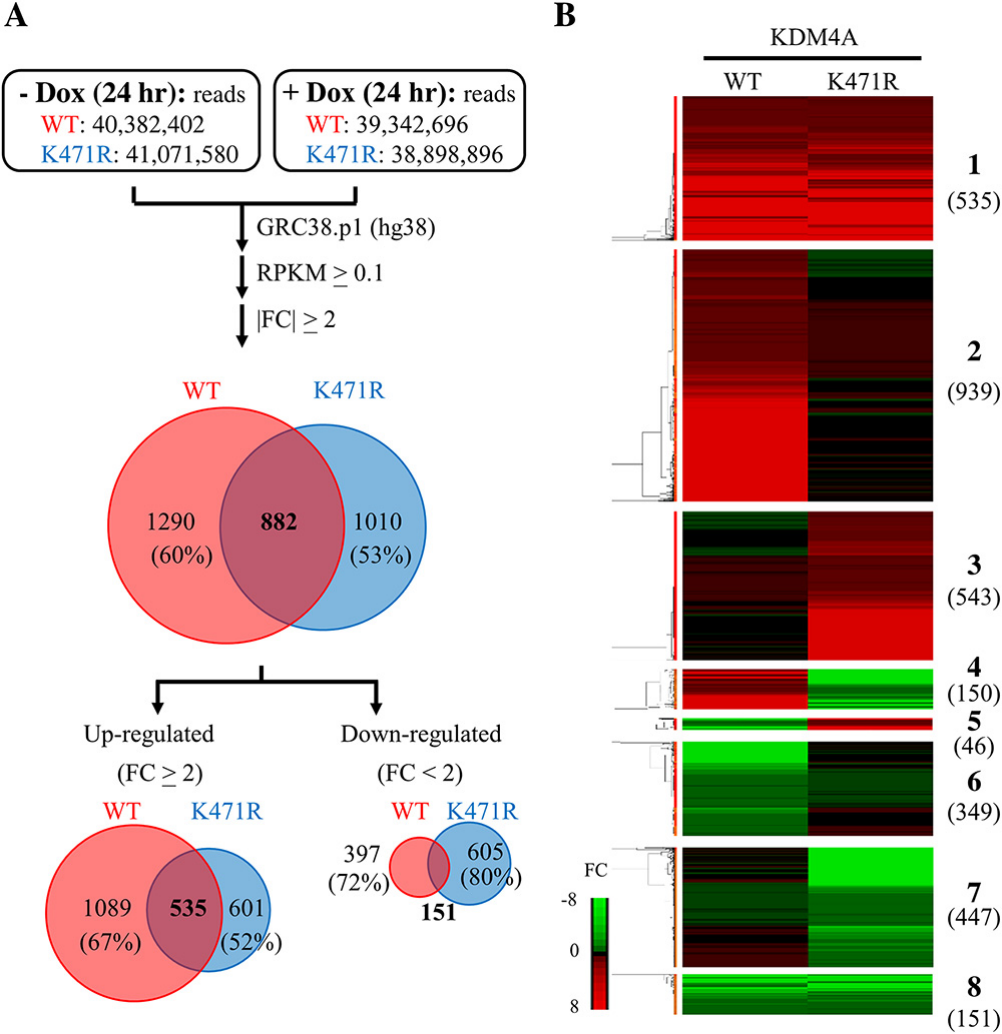
SUMO modification of KDM4A-regulated host genes differentially expressed in KSHV lytic reactivated BCBL-1 cells. (A) Summary of RNA-seq data. TREx-MH-K-Rta-shKDM4A-Flag-KDM4A-WT and -KDM4A-K471R cells were treated with 0.2 μg/mL Dox for 24 h. Cells cultured without any treatment were used as control (Ctrl). Total RNA was extracted and used for RNA-seq on the Illumina HiSeq2000 platform 24 h after treatment. Total reads of each sample were showed in the top boxes. Paired-end reads were aligned to the human reference genome (hg38) using CLC Genomics Workbench 11 (Qiagen) and annotated with RefSeq82 using Partek Genomics Suite 7 (Partek). RPKM greater than 0.1 in any one of the samples was considered as expressed and used for subsequent analysis. Pie chart shows mRNA expression data. The numbers (percentages) of mRNAs that were up- or downregulated more than 2-fold are shown. (B) Heatmap of hierarchical cluster analysis of the mRNA expression data from the treatment conditions described in Fig. 1A.

**FIG 2:**
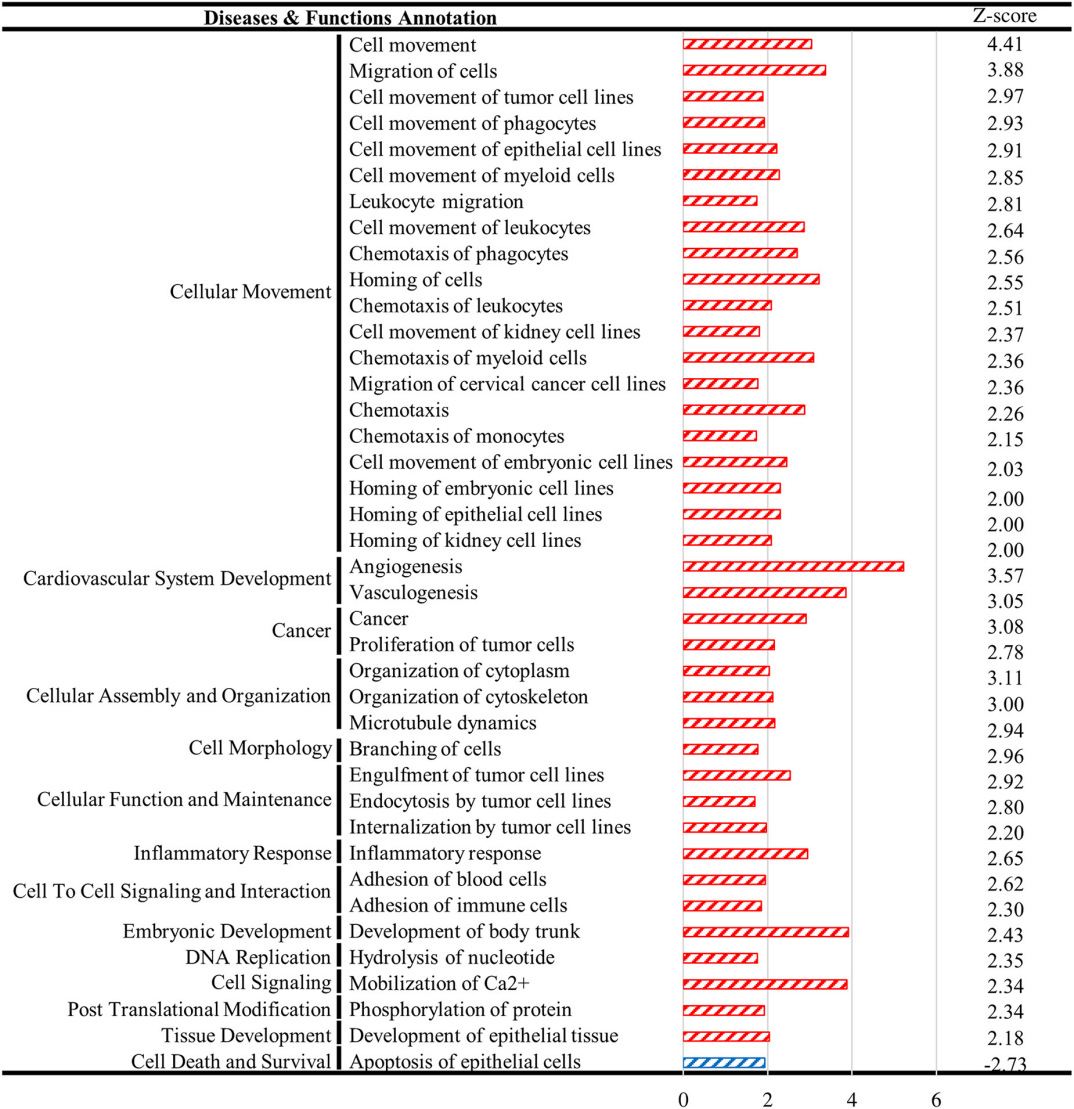
Pathway enrichment of differentially expressed genes (DEGs) between KDM4A-WT and -K471R BCBL-1 after KSHV reactivation. The numbers on the *x* axis indicate -log_10_ *P*-value.

To study how KDM4A SUMOylation influences cellular gene expression in PEL cells after KSHV reactivation, we reanalyzed our RNA-seq data from TREx-MH-K-Rta-shKDM4A-Flag-KDM4A-WT and -K471R BCBL-1 cells (25) focusing on host genes. Consistent with the role of KDM4A in maintaining the “ready to activate” chromatin status, differentially expressed cellular genes of KDM4A-WT and -K471R expressing cells before and after KSHV reactivation showed that more cellular genes were upregulated in KDM4A-WT (1,624) than in KDM4A-K471R (1,136), while more cellular genes were downregulated in KDM4A-K471R (756) when compared with KDM4A-WT (548) (Fig. 1A). Unsupervised hierarchical clustering analysis divided differentially expressed genes (DEGs) upon KSHV reactivation into 8 distinct clusters (clusters 1–8) (Fig. 1B). Clusters 1 and 8 showed KSHV-regulated gene expression changes that did not differ between the two cell lines. Clusters 4 and 5 were genes that were oppositely regulated in the two cell lines after KSHV reactivation. Clusters 2 and 6 comprise genes that were up- and downregulated by KSHV reactivation only in KDM4A-WT cells, respectively. Clusters 3 and 7 comprise genes that were up- and downregulated by KSHV only in KDM4A-K471R cells, respectively. To identify pathways differently regulated by SUMO-modified KDM4A upon KSHV reactivation, the 2,474 genes that were differentially expressed between the two cell lines (clusters 2–7) were subjected to Gene Ontology (GO) analysis using Ingenuity Pathway Analysis (IPA). GO analysis identified a total of 39 activated (z-score > 2) pathways and one inhibited (z-score < −2) pathway (Fig. 2).

### Gene programs regulating cell movement and angiogenesis are activated by KDM4A SUMOylation in KSHV-reactivated PEL.

**FIG 3:**
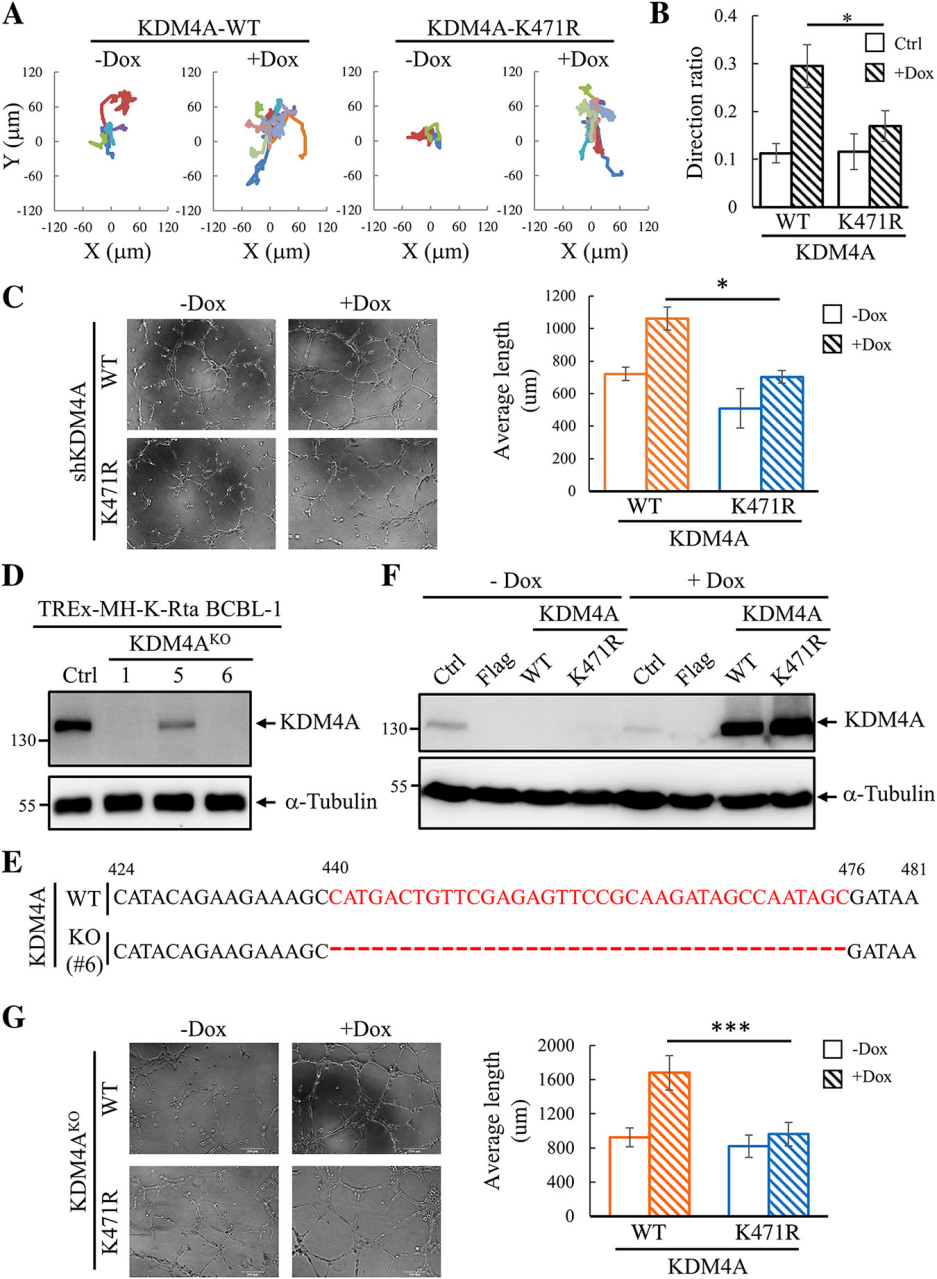
SUMOylation of KDM4A enhanced BCBL-1 cell movement and angiogenesis of HMEC-1 cells. (A) Trajectory tracking of TREx-MH-K-Rta-shKDM4A-Flag-KDM4A-WT and -KDM4A-K471R BCBL-1 cells treated with or without Dox cultivated on top of collagen. The amoeboid cell movement was recorded via a brightfield microscopy for 24 h at 5-min intervals. All the movement cells from 6 microscopic fields were shown. (B) The quantitative data of the directional migration distance was calculated with Image J software. (C) Representative images (×100 magnification) of *in vitro* tube formation assay in HMEC-1 cells treated with conditioned medium (CM) collected from TREx-MH-K-Rta-shKDM4A-Flag-KDM4A-WT and -KDM4A-K471R BCBL-1 cells treated with or without Dox (0.2 μg/mL) for 72 h (left panel). Quantification of tube length (right panel). (D) Generation of KDM4A knockout TREx-MH-K-Rta BCBL-1 cell line by CRISPR/Cas9 system. Immunoblotting of KDM4A in different TREx-MH-K-Rta BCBL-1 knockout clones. (E) The knockout clone (#6) was confirmed by Sanger sequencing of PCR amplicons. (F) Immunoblotting of KDM4A expression in the control (Ctrl), Flag-tag-expressing vector (Flag), KDM4A-WT, and KDM4A-K471R transduced TREx-MH-K-Rta-KDM4A^KO^ BCBL-1 cells with or without Dox (0.2 μg/mL) treatment for 72 h. (G) Representative images (×100 magnification) of *in vitro* tube formation assay in HMEC-1 cells treated with CM collected from TREx-MH-K-Rta-KDM4A^ko^-Flag-KDM4A-WT and -KDM4A-K471R BCBL-1 cells treated with or without Dox (0.2 μg/mL) for 72 h (left panel). Quantification of tube length (right panel).

Interestingly, GO analysis revealed that cell movement, cardiovascular system development, cancer, and cellular assembly and organization are the major activation pathways (Fig. 2). The amoeboid movement has been long known as a main movement method for T lymphocytes and hematopoietic cells (32). Moreover, a recent report revealed the impact of amoeboid movement on the dissemination of diffuse large B-cell lymphoma (DLBCL), an aggressive lymphoid malignancy (31). According to GO analysis, we first determined whether KDM4A enhances B cell amoeboid movement using a 3-dimensional (3D) timelapse assay. Trajectory tracking showed that KSHV reactivation elicited a marked increase in B cell movement in KDM4A-WT, but not KDM4A-K471R, expressing BCBL-1 cells (Fig. 3A and B). This finding confirmed the participation of KDM4A in movement and possibly in the dissemination of KSHV-infected B cells.

Cytokines are important mediators of angiogenesis. Since GO analysis showed enrichment in angiogenesis pathways, we first analyzed whether conditioned medium (CM) from BCBL-1 cells could enhance endothelial cell angiogenesis. As expected, CM from KSHV-reactivated TREx-MH-K-Rta-shKDM4A-Flag-KDM4A-WT BCBL-1 cells induced a slight (<1.5-fold change) but significantly higher angiogenesis in HMEC-1 when compared with CM from TREx-MH-K-Rta-shKDM4A-Flag-KDM4A-K471R cells (Fig. 3C). We suspect the small difference in angiogenesis is likely due to the remaining endogenous KDM4A. To confirm this, we generated the KDM4A knockout-rescue BCBL-1 cell lines inducibly expressing KDM4A-WT or KDM4A-K471R (Fig. 3D–3F) and performed the angiogenesis assay. Indeed, CM from KDM4A^KO^ cell line complemented with KDM4A-WT, but not KDM4A-K471R, induced a significantly higher angiogenesis (~2-fold) in HMEC-1 following KSHV reactivation (Fig. 3G). These data indicate that cellular factors in CM contributed to the observed angiogenesis.

### Interleukin-10 (IL-10) is a novel KDM4A target gene participating in modulating angiogenesis function of PEL cells.

**FIG 4:**
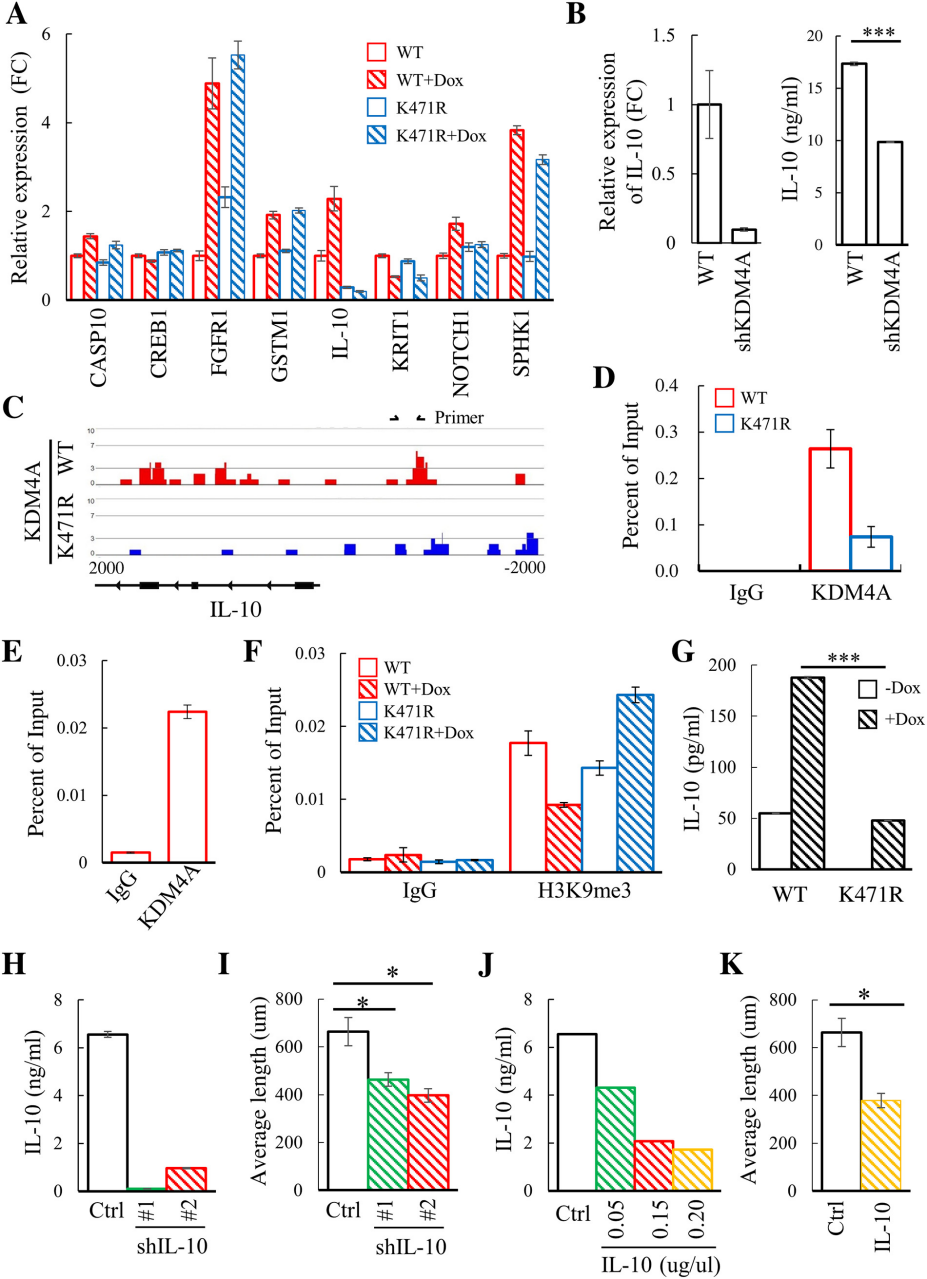
Identification of IL-10 as a novel target gene of KDM4A potentially involved in angiogenesis of endothelial HMEC-1 cells. (A) Real-time reverse transcriptase-quantitative PCR (RT-qPCR) analysis of the expression of angiogenesis-related genes in TREx-MH-K-Rta-shKDM4A-Flag-KDM4A-WT and KDM4A-K471R cells treated with Dox (0.2 μg/mL) for 0 and 24 h. The results are expressed as fold change (FC) compared to KDM4A-WT cells without Dox (assigned a value of 1). (B) The expression of IL-10 in the TREx-MH-K-Rta (WT) and TREx-MH-K-Rta-shKDM4A BCBL-1 cells was analyzed by RT-qPCR (left panel). Culture supernatants were collected, and levels of IL-10 were measured by ELISA (right panel). The results are expressed as FC compared to wild-type (WT) cells (assigned a value of 1). (C) Histograms of ChIP-seq profiles for KDM4A binding at IL-10 loci in TREx-MH-K-Rta-shKDM4A-Flag-KDM4A-WT and -KDM4A-K471R BCBL-1 cells. (D and E) ChIP assay was performed with chromatin prepared from TREx-MH-K-Rta-shKDM4A-Flag-KDM4A-WT and -KDM4A-K471R BCBL-1 cells (D) and from TREx-MH-K-Rta BCBL-1 cells (E) using rabbit IgG and anti-KDM4A antibody. ChIP DNA was quantified by RT-qPCR using primer pairs specific for promoter regions of IL-10. (F) ChIP-qPCR assay was performed on noninduced and Dox-induced TREx-MH-K-Rta-shKDM4A-Flag-KDM4A-WT and -KDM4A-K471R BCBL-1 cells using rabbit IgG and anti-H3K9me3 antibody. ChIP DNA levels were determined as described in (D) and (E). (G) Culture supernatants from TREx-MH-K-Rta-shKDM4A-Flag-KDM4A-WT and KDM4A-K471R cells with or without Dox (0.2 μg/mL) treatment for 72 h were collected and the levels of IL-10 were measured by ELISA. (H) TREx-MH-K-Rta BCBL-1 cells were transient transduced with lentivirus overexpressing shIL-10 (clone #1 and #2). Culture supernatants from the KSHV-reactivated control (Ctrl) and IL-10 knockdown (shIL-10) BCBL-1 cells were collected, and IL-10 levels were determined by ELISA. (I) Quantification of the tube length of HMEC-1 treated with CM collected from cells treated as described in (H). (J) IL-10 levels in KSHV-reactivated BCBL-1 cells with or without preincubation with 0.05, 0.15, and 0.2 μg/mL IL-10 nAb for 10 min at 25°C were determined by ELISA. (K) Quantification of the tube length of HMEC-1 treated with CM collected from TREx-MH-K-Rta BCBL-1 cells with or without preincubation with 0.2 μg/mL IL-10 nAb for 10 min at 25°C.

To identify the SUMOylated KDM4A target gene responsible for angiogenesis, genes differentially activated in TREx-MH-K-Rta-shKDM4A-Flag-KDM4A-WT and KDM4A-K471R BCBL-1 cells after KSHV reactivation in the angiogenesis pathway were analyzed by RT-qPCR. Among them, IL-10, an immunosuppressive cytokine contributing to viral persistence in and pathogenesis of KSHV-associated lymphoproliferative disorders (33–35), showed a consistent induction by KSHV reactivation (>1.5-fold) in KDM4A-WT-, but not in KDM4A-K471R-expressing cells (Fig. 4A). To show that KDM4A indeed regulates IL-10 expression, IL-10 level was further measured in KDM4A knockdown TREx-MH-K-Rta-shKDM4A BCBL-1 cells compared to wild-type cells. Consistently, knockdown of KDM4A (Fig. 4B, left panel) reduced IL-10 expression and secretion (Fig. 4B). These data indicated that IL-10 is a direct target of KDM4A.

We have previously shown that SUMOylation stabilizes KDM4A binding on the KSHV genome and enables viral gene transactivation (25). To determine whether SUMOylation is also involved in maintaining KDM4A binding on the host genome, we performed a chromatin immunoprecipitation sequencing (ChIP-seq) experiment of KDM4A using TREx-MH-K-Rta-shKDM4A-Flag-KDM4A-WT and -KDM4A-K471R BCBL-1 cells (Fig. S1A). Consistent with our previous finding observed in viral genomes, significantly higher chromatin binding of KDM4A-WT was identified near transcription start sites (TSSs) of host genes (Fig. S1B and S1C). A Venn diagram of peak calling from MACS2 shows the loss of around 80% of KDM4A binding peaks in KDM4A-K471R (Fig. S1D). These results demonstrate that SUMOylation is also required for stabilizing the binding of KDM4A on promoter regions of host genes. Importantly, our ChIP-seq data showed that KDM4A-WT, but not KDM4A-K471R, binds to the proximal promoter of IL-10 (Fig. 4C). The enrichment of KDM4A-WT, but not KDM4A-K471R, binding at the proximal promoter region of IL-10 was confirmed by ChIP-qPCR analysis (Fig. 4D). Moreover, the binding of endogenous KDM4A on the IL-10 promoter was confirmed in BCBL-1 (Fig. 4E). Since our previous reports showed that KDM4A prevents H3K9me3 accumulation on the KSHV genome during reactivation and is required for proper lytic gene expression (25, 27), we further detected H3K9me3 on the IL-10 promoter in both KSHV latent infected and lytic reactivated BCBL-1 cells expressing KDM4A-WT and KDM4A-K471R. Consistently, H3K9me3 level on the IL-10 promoter was reduced in KDM4A-WT-, but not KDM4A-K471R-expressing BCBL-1 cells after KSHV reactivation (Fig. 4F). Altogether our data indicate that KDM4A binding on the IL-10 promoter enabling its transcription.

To determine whether IL-10 could be one of the active factors secreted by BCBL-1 cells that enhances endothelial cell angiogenesis, we measured IL-10 levels in the CM of KDM4A-WT- and KDM4A-K471R-expressing cells using ELISA. Consistently, KSHV reactivation induced IL-10, and its level was higher in both KSHV latent infected and lytic reactivated BCBL-1 cells expressing KDM4A-WT when compared with KDM4A-K471R (Fig. 4G). Next, we knocked down IL-10 in BCBL-1 cells using the short hairpin RNA (shRNA) approach. The successful knockdown of IL-10 by two shRNAs was first confirmed by ELISA (Fig. 4H). As expected, IL-10 knockdown significantly reduced the angiogenesis of HMEC-1 (Fig. 4I). We further verified the angiogenesis-promoting effect of IL-10 by using IL-10 neutralizing antibody (NAb). To this end, the neutralizing efficacy was first tested by incubating CM from KSHV-reactivated BCBL-1 cells with 0.05, 0.15, and 0.2 μg/mL of IL-10 NAb, followed by analyzing IL-10 levels using ELISA (Fig. 4J). Next, we treated HMEC-1 cells with CM from KSHV-reactivated BCBL-1 cells in the presence or absence of 0.2 μg/mL IL-10 NAb. Again, IL-10 NAb was sufficient to inhibit the angiogenesis effect of CM from KSHV-reactivated BCBL-1 cells (Fig. 4K). Altogether, we conclude that IL-10 is a downstream target of KDM4A that participated in PEL cell-mediated promotion of endothelial cell angiogenesis.

### KDM4A SUMOylation decides the fate of PEL cells after KSHV reactivation.

**FIG 5:**
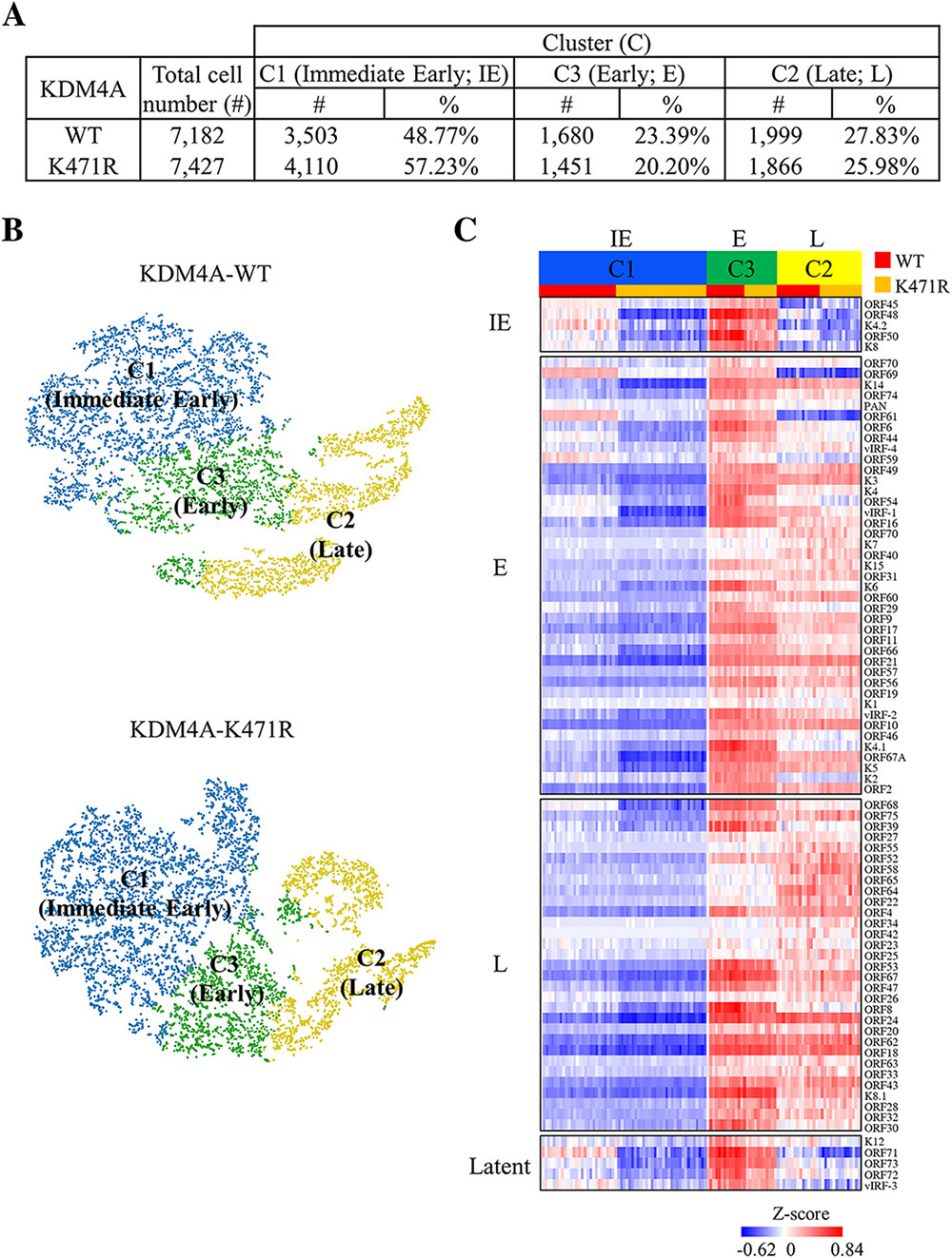
Dissection of KSHV lytic reactivation stages in TREx-MH-K-Rta-shKDM4A-Flag-KDM4A-WT and -KDM4A-K471R BCBL-1 cell lines treated with Dox (0.2 μg/mL) for 24 h. (A) The number (cell #) and percentage (%) of TREx-MH-K-Rta-shKDM4A-KDM4A-WT and -KDM4A-K471R BCBL-1 cells in single-cell RNA sequencing (scRNA-seq). (B) T-distributed stochastic neighbor embedding (t-SNE) plot demonstrating cells in different lytic stages (colored and labeled by their marker genes). (C) The heatmap shows the expression (standardized average count) of KSHV lytic genes in each cluster. Row: KSHV genes. Column: cells.

As GO analysis showed that SUMOylated KDM4A is negatively associated with cell death, the role of KDM4A in protecting against KSHV reactivation-induced cell death was further evaluated. Since KSHV reactivation by no means guarantees that all virally infected cells undergo a similar cell fate (36), we therefore performed a single-cell RNA sequencing (scRNA-seq) analysis to evaluate the status of PEL cells in response to KSHV reactivation. A total of 7,182 and 7,427 qualified cells with an average of 4,604 and 4,461 gene features detected per cell in doxycycline (Dox)-treated TREx-MH-K-Rta-shKDM4A-KDM4A-WT (Table S1) and -KDM4A-K471R (Table S2) samples, respectively, were obtained and applied for further analysis (Fig. 5A). Cell composition was first analyzed by unsupervised k-means clustering using all genes detected (including cellular and viral genes) and visualized by t-SNE (Fig. 5B). Three clusters were defined. Next, a standardized expression value of viral genes in each cell was plotted by heatmap using Partek Flow. Based on the expression kinetics, KSHV lytic gene was divided into immediate early (IE), early (E), and late (L) genes. Interestingly, in KDM4A-WT-expressing cells (Fig. 5C), cluster 1 (C1) expressed mostly viral IE genes and some E genes, indicating that cells in C1 represents the initiation of KSHV reactivation. For cluster 3 (C3), all viral IE and E genes and around half of L genes in KDM4A-WT-expressing cells were expressed at a relatively high level (Fig. 5C), representing vigorous viral replication. For cluster 2 (C2), most IE genes and some E genes in KDM4A-WT-expressing cells were expressed at a relatively low level. Moreover, almost all L genes in KDM4A-WT-expressing cells were expressed in a relatively high level (Fig. 5C), indicating that cells in C2 represent the late stage of KSHV reactivation. Altogether, according to KSHV lytic gene expression, C1, C3, and C2 were defined as IE, E, and L phases of the lytic cycle, respectively (Fig. 5C). Consistent with our previous findings that KDM4A SUMOylation is required for KSHV lytic reactivation (25), the viral gene expression (Fig. 5C) and the cell population percentage in E (C3) and L (C2) phase (Fig. 5A) are generally higher in KDM4A-WT than in KDM4A-K471R at the single-cell level.

**FIG 6:**
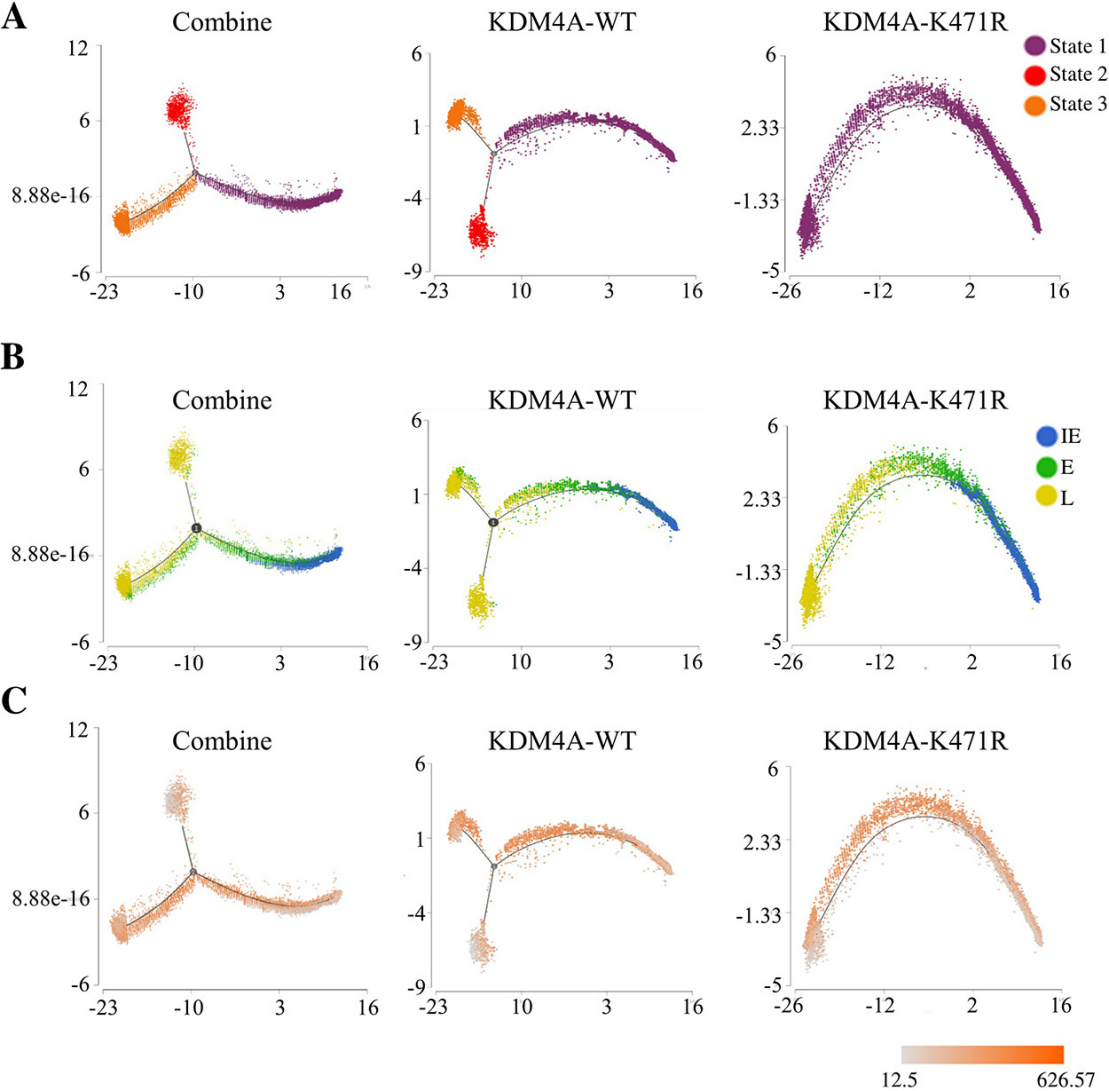
Trajectory analysis of TREx-MH-K-Rta-shKDM4A-Flag-KDM4A-WT and -KDM4A-K471R BCBL-1 cells. The trajectory plot of combined (left panel), TREx-MH-K-Rta-shKDM4A-KDM4A-WT (middle panel), and -KDM4A-K471R (right panel) BCBL-1 cells. (A) The trajectory plots are colored with purple, red, and orange, indicating states 1, 2, and 3, respectively. (B) The trajectory plots are colored with blue, green, and yellow, indicating immediate early (IE), early (E), and late (L), respectively. (C) The trajectory plots are colored with the expression of KSHV genes.

Next, we performed a trajectory analysis using combined scRNA-seq data from TREx-MH-K-Rta-shKDM4A-Flag-KDM4A-WT and -K471R BCBL-1 cells. Three states were defined (Fig. 6A, left panel). When the trajectory analysis was performed individually in KDM4A-WT- and K471R-expressing cell lines, a similar result was found in cells expressing KDM4A-WT (Fig. 6A, middle panel), while the KDM4A-K471R mutant lost state 3 (Fig. 6A, right panel). The loss of state 3 in the SUMO-deficient mutant suggests the potential role of KDM4A SUMOylation in regulating cell destiny after KSHV reactivation. To determine the KSHV reactivation status in the trajectory, we first colored the cells by their cluster assignments (Fig. 5B) that represent different phases of the KSHV lytic cycle onto the trajectory plots (Fig. 6B). In the combined trajectory, C1 (IE), C3 (E), and C2 (L) were all found in state 1, while C3 (E) was split into state 1 and state 3 and C2 (L) was shown in both state 2 and state 3 (Fig. 6B, left panel). Again, the KDM4A-WT cell line showed a similar pattern as in the combined analysis (Fig. 6B, middle panel), while KDM4A-K471R showed a sequential distribution of C1 (IE), C3 (E), and C2 (L) along the trajectory (Fig. 6B, right panel). Our data indicate that PEL cells undergo different cell fates in the late phase of KSHV reactivation. Next, the expression levels of viral genes were plotted onto each cell in the trajectory. Importantly, cells in states 1 and 3 express comparable levels of viral genes, while cells in state 2 express relatively lower levels of viral genes (Fig. 6C). The result indicated that although cells in both states 2 and 3 express late KSHV lytic cycle genes, only cells in state 3 are associated with robust KSHV transcription. The loss of state 3 in the KDM4A-K471R mutant (Fig. 6A) supports the role of SUMOylated KDM4A in maintaining KSHV gene expression in PEL cells undergoing viral reactivation (25).

### KDM4A protects KSHV-reactivated PEL cells from death.

**FIG 7:**
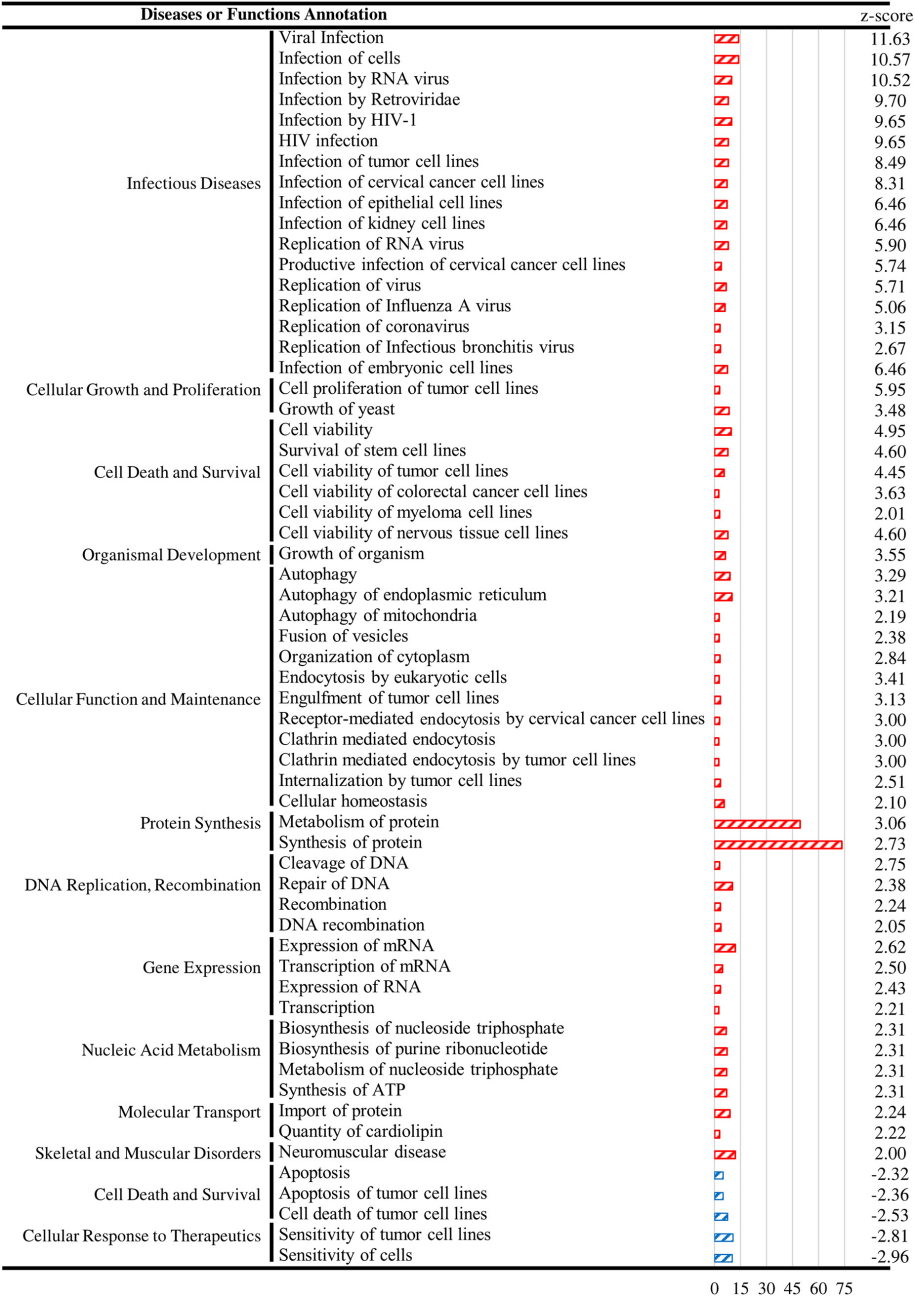
Pathway enrichment of DEGs between states 2 and 3 of the trajectory. The numbers of *x* axis indicate -log_10_ *P*-value.

The split of C2 (L) into states 2 and 3 of the trajectory suggests virally infected cells undergo different fates in response to viral reactivation. Elucidating the differences between these two states may help reveal cell fate decisions of lytic KSHV-infected cells. To this end, the DEGs between these two states (state 3 versus state 2; Table S3) in the combined trajectory (Fig. 6A, left panel) were subjected to GO analysis using IPA. The analysis identified a total of 47 activation (z-score > 2) and 5 inhibition (z-score < -2) pathways (Fig. 7). Consistently, the identified activation pathways were mainly involved in infectious disease, DNA replication, cell proliferation, and survival, indicating state 3 were cells that survived in robust KSHV lytic replication. Moreover, cell death was identified as the major inhibitory pathway (Fig. 2), demonstrating that cells in state 3 tend toward anticell death. The tendency to go toward state 2 and the loss of state 3 in cells expressing KDM4A-K471R (Fig. 6A, right panel) indicates that the loss of KDM4A tends to induce cell death in PEL cells at the late stage of KSHV reactivation. In line with our previous report showing that SUMO modification of KDM4A is required for lytic gene activation during viral reactivation (25), the tendency of KDM4A-K471R-expressing cells to go toward state 2 may be due to induction of apoptosis with less expression of the viral lytic antiapoptotic genes.

**FIG 8:**
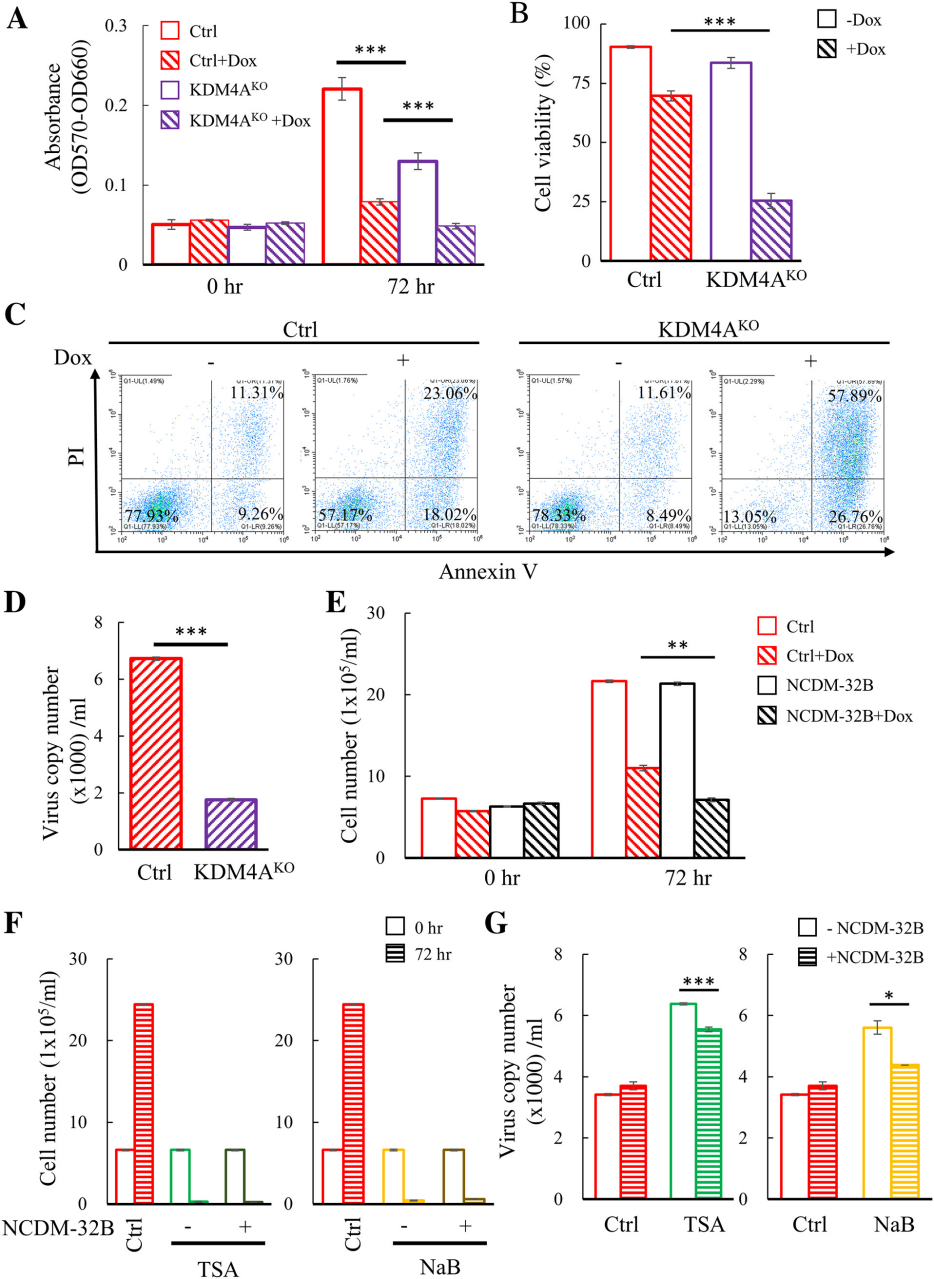
KDM4A is required for the survival and virus production of BCBL-1 cells after KSHV reactivation. (A) Proliferation of TREx-MH-K-Rta and TREx-MH-K-Rta KDM4A knockout (TREx-MH-K-Rta-KDM4A^KO^) BCBL-1 cell lines before after Dox treatment for 72 h were assessed by MTT assay. (B) Viable cells treated as described in (A) were counted by trypan blue dye exclusion assay using Countess 3 FL. (C) TREx-MH-K-Rta and TREx-MH-K-Rta-KDM4A^KO^ cells were treated with Dox (0.2 μg/mL) for 0 and 72 h, followed by the stain of annexin V and propidium iodide using an apoptotic detection kit. The fluorescence intensity of the stained cells was analyzed using fluorescence-activated cell sorting (FACS). Cells without staining were used as negative control. (D) Supernatants from TREx-MH-K-Rta and KDM4A^KO^ BCBL-1 cells treated as described in (A) for 72 h were collected, filtered, and the viral titers were determined by analyzing the virion-associated DNA levels using TaqMan qPCR. (E) TREx-MH-K-Rta BCBL-1 cells were treated with 0.2 μg/mL Dox, 60 μM NCDM-32B, or both for 0 and 72 h. Cell numbers were counted by trypan blue dye exclusion assay using Countess 3 FL. (F) TREx-MH-K-Rta BCBL-1 cells were treated with 500 ng/mL TSA, 1 mM NaB, 60 μM NCDM-32B/500 ng/mL TSA or 60 μM NCDM-32B/1 mM NaB. Cell numbers were counted as described in (E). Cells without treatment used as a control (Ctrl). (G) Supernatants from TREx-MH-K-Rta BCBL-1 cells treated as described in (F) were collected and filtered, and the viral titers were determined as described in (D). (Data represent mean±SEM; *n* = 3; *, *P* < 0.05; ***, *P* < 0.005).

The success of inducing KSHV reactivation to treat PEL is largely dependent on concomitantly eliciting cell death (18) and preventing infectious virus production (17). Taken together with our previous report showing that KDM4A is required for KSHV production, we hypothesize that loss of KDM4A may result in cell death and loss of viral production. Therefore, we further evaluate cell viability and viral particle production in parental and TREx-MH-K-Rta-KDM4A^KO^ BCBL-1 cells after KSHV reactivation. Consistent with our hypothesis, the results showed that compared with the control, knockout of KDM4A significantly reduced cell number (Fig. 8A) and cell viability (Fig. 8B) after KSHV reactivation. The reduction of cell number and cell viability is likely due to cell apoptosis (Fig. 8C). Moreover, consistent with our previous report showing that knockdown of KDM4A resulted in the reduction of viral production (~50–60%) (27), the viral production was significantly reduced in TREx-MH-K-Rta-KDM4A^KO^ BCBL-1 cells when compared with the control (~70–80%) (Fig. 8D). These results suggest that inhibition of KDM4A may be a potential therapeutic approach to treat PEL. To study this, we treated the control and Dox-induced TREx-MH-K-Rta BCBL-1 cells with or without the KDM4A inhibitor NCDM-32B. Indeed, NCDM-32B treatment resulted in a slight but significant reduction of cell number after KSHV reactivation (Fig. 8E). Next, we treated TREx-MH-K-Rta BCBL-1 cells with the HDAC inhibitor Trichostatin A (TSA) or sodium butyrate (NaB) together with NCDM-32B. The treatment of TSA and NaB resulted in severe cell death, we were unable to see the cell number difference in the presence and absence of NCDM-32B. However, a slight but significant reduction of viral production was observed with the combination of HDAC and KDM4A inhibitors. These data together suggest that inhibition of KDM4A or its SUMO modification may potentiate current PEL therapy.

## DISCUSSION

The biphasic life cycle of KSHV is tightly epigenetically regulated (37). Therefore, HDAC inhibitor TSA and NaB have long been used to induce KSHV reactivation (16) and have been proposed as potential treatments for PEL (17, 18, 38). However, the therapeutic effects of other histone modifications, particularly the application of histone methylation, are also worth evaluating. KDM4A, also known as Jumonji C domain-containing histone demethylase 2A (JMJD2A), is the first identified histone trimethyl lysine demethylase that removes heterochromatin mark H3K9me3, creating an open chromatin environment, and thus allowing transcription activation (39). Our previous report showed that KDM4A binds to latent KSHV genome regions that lack H3K9me3 and is required for proper KSHV lytic replication and virion production (25, 27). Moreover, we found that KDM4A is SUMO-modified at K471. Since SUMOylation is required for chromatin binding and consequently demethylase activity of KDM4A, rescuing overexpression of KDM4A-K471R mimics the knockdown phenotype (25). In addition to the viral effect, KDM4A has also been recognized as an oncogene that is highly expressed in various human cancers (40, 41) and inhibits apoptosis (42–44). Therefore, we hypothesize that induction of KSHV reactivation in KDM4A knockdown BCBL-1 cells or cells expressing a SUMO-deficient mutant of KDM4A may lead to incomplete viral lytic replication and cell death. If this is true, KDM4A can be considered as a promising antioncogenic virotherapy.

Although induced lytic reactivation of KSHV from latently infected B lymphoma cells can be a potential therapy for PEL, KSHV encodes viral genes that inhibit apoptosis in both latent and lytic infection, hampering this therapy. During latent infection, inhibition of apoptosis is important to maintain persistent infections. In lytic reactivation, KSHV inhibits apoptosis to prevent cell death before viral replication and release (45). Therefore, simultaneously inducing KSHV reactivation and inhibiting antiapoptosis has been widely studied for the treatment of PEL; this included the use of nutlin-3 (24, 46), cisplatin (47, 48), silver nanoparticle (nAg) (49), cyclin-dependent kinase 1 (Cdk1) inhibitor (50), and histone deacetylase (HDAC) inhibitor (17, 18, 38). The success of these approaches mainly relies on the activation of p53 (17, 18, 24, 46, 48) or induction of oxidative stress (47, 49). However, the mutual inhibition between KSHV lytic reactivation and apoptosis was revealed as virus replication predominately observed in the nonapoptotic subpopulation of PEL cells (24, 38, 50). Therefore, cell death signaling in response to viral reactivation can only be answered at a single-cell level. To this end, we performed scRNA-seq analysis in KSHV-reactivated BCBL-1 cells. Trajectory analysis of scRNA-seq revealed two distinct cell states in the late lytic phase of KSHV reactivations (Fig. 6); one expressed high levels of viral gene showing less apoptotic signaling, while another state expressed that low levels of viral genes are apoptotic cells. The loss of less apoptotic state in SUMO-deficient KDM4A (Fig. 6) supports the notion of inhibiting apoptosis by KDM4A after KSHV reactivation. Consistently, GO analysis showed that KDM4A plays a key role in maintaining KSHV replication and cell survival after viral reactivation (Fig. 7).

Unexpectedly, bioinformatic analyses of bulk RNA-seq data revealed that KDM4A also participates in the regulation of cell movement and angiogenesis, thus possibly contributing to the dissemination of PEL. PEL is a lymphomatous effusion localized in body cavities, and this tropism depends on lymphocyte migration. Our data here suggest that targeting KDM4A may interrupt PEL homing. In addition, a previous report showed that angiogenic factor vascular endothelial growth factor (VEGF) secreted by PEL cells interacted with endothelial cells, induced angiogenesis, and contributed to the invasive behavior of PEL invasion (30). Here, our integrated ChIP-seq and RNA-seq analysis identified IL-10 as a novel direct target of KDM4A (Fig. 4), and we showed that IL-10 secreted from KSHV-reactivated BCBL-1 cells promotes angiogenesis (Fig. 3 and 4). Since immune cytokines are important mediators for angiogenesis, our data here represent that IL-10 may be a novel target for interrupting the progression of PEL.

Importantly, IL-10 is a multifunctional cytokine that regulates cell growth and differentiation, immune response, and tumor development, particularly in hematopoietic neoplasms (33, 51, 52). Moreover, a specific subset of regulatory B cells termed B10 cells that produced IL-10 was recently found to inhibit immunotherapy-mediated clearance of non-Hodgkin’s B lymphoma cells (53). In addition to inhibiting immunotherapy, the secretion of IL-10 by PEL has also been shown to confer immune escape (54) and drug resistance (55). One potential explanation is that IL-10 works in autocrine/paracrine loops to induce the antiapoptotic effect in non-Hodgkin’s B-cell lymphoma (56, 57). Although the transcriptional regulation of IL-10 production in immune cells, including T cells, dendritic cells (DCs), and macrophages, has been widely studied (58), little is known about the regulation of IL-10 expression in B lymphoma cells. Here, we show that KDM4A binds to the proximal promoter of IL-10 in PEL cells and regulates IL-10 production after KSHV reactivation (Fig. 4); our data indicated that KDM4A epigenetically regulated IL-10 production in KSHV-infected PEL cells. Identification of an apoptosis pathway inhibited by KDM4A after KSHV reactivation (Fig. 2 and 7) suggested that IL-10 may also be a potential underlying mechanism that links KDM4A to protection against cell death. Moreover, IL-10 has also been linked to enable the virus to evade host immunity and establish persistent infection (59). In KSHV, elevated levels of IL-10 are believed to contribute to KSHV persistence in and pathogenesis of KSHV-associated lymphoproliferative disorders (33, 34). Altogether, the production of IL-10 by KSHV-reactivated PEL cells might facilitate PEL dissemination through inducing cell migration and angiogenesis, inhibition of immunity, apoptosis, and virus propagation. Therefore, inhibition of KDM4A may have an additional beneficial antitumor effect *in vivo* that is worth further investigation in future research.

PEL is a lymphomatous effusion localized in body cavities without tumor mass that cannot be surgically removed. Since the current CHOP treatment efficiency is restricted, finding new therapeutic approaches for PEL remains a challenge. Targeting dominant viruses with drugs that reactivate KSHV lytic replication is an attractive option for treating KSHV-associated hematologic malignancy, such as PEL. However, the therapeutic effectiveness of current lytic induction therapies in PEL has yet to be improved. Therefore, therapeutic candidates that can selectively target KSHV lytic replicated PEL cells for death without increasing viral load need to be identified. In this study, we demonstrate that KDM4A is involved in protecting PEL cells from death, PEL cell migration, and angiogenesis induced by viral reactivation. Most importantly, we identified IL-10 as a novel target gene of KDM4A. Together with our previous findings, inhibition of KDM4A or its SUMOylation appeared to achieve a desirable clinical goal of concomitantly inducing incomplete viral lytic replication that does not increase viral load, increases cell death, and prevents PEL dissemination.

## MATERIALS AND METHODS

### Cell culture and cell line construction.

The doxycycline (Dox)-inducible TREx-MH-K-Rta BCBL-1 cell line, with Myc-His-tagged K-Rta, was maintained in RPMI 1640 containing 15% FBS, 20 μg/mL blasticidin, and 200 μg/mL hygromycin (Invitrogen, 10687010). The doxycycline (Dox)-inducible TREx-MH-K-Rta-shKDM4A BCBL-1 cell line was maintained as described for TREx-MH-K-Rta BCBL-1 and supplemented with 1 μg/mL puromycin (Invitrogen). TREx-MH-K-Rta-shKDM4A-Flag-KDM4A-WT and TREx-MH-K-Rta-shKDM4A-Flag-KDM4A-K471R BCBL-1 were maintained as described for TREx-MH-K-Rta-shKDM4A BCBL-1 cells and supplemented with 100 μg/mL G418 (AMRESCO).

KDM4A knockout TREx-MH-K-Rta-KDM4A^KO^ BCBL-1 cells were generated by transfected TREx-MH-K-Rta BCBL-1 cells with the plasmid PX330 that expresses guide RNA (gRNA, 5′-CCG CAA GAT AGC CAA TAG CGATA-3′) targeting exon 3 of KDM4A and Cas9 using X-tremeGENE HP (Roche, 6366236001). Three days after transfection, RFP-positive cells were isolated by fluorescence-activated cell sorting (FACS) and plated in 96-well plates at 1 cell/well. The expression of KDM4A was analyzed by immunoblotting assays. PCR fragments (PCR primer sequence: 5′-CTT GCC AGG TTT CTC ATC TGT C-3′ and 5′-GGT TTC CAC TCA CTT ATC GCT-3′) amplified from genomic DNA extracted from the 6th clone (#6) of TREx-MH-K-Rta-KDM4A^KO^ BCBL-1 cells were subjected to Sanger sequencing.

293T was cultured in Dulbecco’s modified Eagle’s medium (DMEM, Gibco, 12100-038) containing 10% FBS (Gibco, 26140-079). Human dermal microvascular endothelial (HMEC1) cell line was cultured in MCDB131 medium (Gibco, 2027-357) supplemented with 10% FBS, 1% l-glutamine, 20 ng/mL EGF (PRO-SPEC, cyt-217), and 5 μg/mL hydrocortisone. All cells used in this study were cultured at 37°C and supplied with 5% CO_2_.

### Expression vectors.

IL-10 knockdown plasmids (#1, TRCN0000058458 and #2, TRCN0000372522) were purchased from RNA Technology and Gene Manipulation Core in Academia Sinica. pLenti4-CMV/TO-KDM4A-WT and -KDM4A-K471R were used to ectopically express KDM4A in TREx-MH-K-Rta-KDM4A^KO^ BCBL-1 cells.

### Single-cell RNA sequencing (scRNA-seq) and data processing.

The single-cell suspension with viability >95% was loaded onto the 10x Genomics Single-Cell-A Chip for target capture of ~8,000 cells/chip. The cDNA library was prepared using Chromium Next GEM Single Cell 3′ Reagent Kits v3.1 (10x Genomics, San Francisco, CA, USA; catalog no. PN-1000121), according to the manufacturer’s protocol. The library was sequenced by NovaSeq 6000 (Illumina, San Diego, CA, USA; catalog no. 20012850) at a depth of ~150 M reads per sample. The raw sequencing data were processed by Cell Ranger (10x Genomics, San Francisco, CA, USA; version 3.1.0) analysis pipeline and aligned with the human reference genome (GRCh38) and the HHV-8 reference genome (NC_009333.1). Cells with low-quality cell barcodes or with a high percentage of reads mapping to the mitochondrial genome were excluded. A total of 7,172 and 7,427 cells from TREx-MH-K-Rta-shKDM4A-Flag-KDM4A-WT and -K471R BCBL-1, respectively, were included for clustering analysis using k-means model and visualized by t-distributed stochastic neighbor embedding (t-SNE). All genes in TREx-MH-K-Rta-shKDM4A-Flag-KDM4A-WT and -K471R BCBL-1 cells were used to construct the pseudotime trajectory using Partek Flow (Partek, Louis, MO, USA; catalog no. 4485102). DEGs were identified using Partek Flow (Partek), filtered by average read >1 (|log_2_ fold change| ≥ 1) and *P*-value < 0.05, and then subjected to GO analysis using ingenuity pathway analysis (IPA) software (Qiagen, Hilden, Germany).

### Trajectory tracking of BCBL-1 cell migration.

1 × 10^6^ BCBL-1 cells were suspended overnight in 200 μL of 1.5 mg/mL collagen solution (Advanced BioMatrix Inc, Carlsbad, CA). Cells were observed for 24 h in a humidified CO_2_-equilibrated chamber with a Nikon Eclipse Ti microscope (Nikon Inc, Japan) equipped with a motorized stage (Mikon, Japan). The images were captured every 5 min. For the reactivation experiments, 0.2 μg/mL Dox was added the day before tracking. The movement of cells in each group was tracked for 279 frames. All movement cells in the captured fields were selected by trajectory tracking with ImageJ software. Measurements of a single cell trajectory were produced by 279 displacements, and all displacements from the same cell line were used to plot the histograms. Results were displayed with box and whisker plots.

### Tube formation assays.

Basement membrane extract (BME) (Trevigen, 3432-010-01) was coated in 96-well plates (50 μL/well) for 1 h at 37°C. Then, 2 × 10^4^ HMEC-1 in MCBD131 medium (100 μL/well) were placed onto the BME and incubated for 6 h at 37°C. The tube structures were captured by an inverted light microscope (100×). The average tube length in each field was measured by ImageJ. For each treatment, the tube length was calculated using the average of 10 microscopic fields.

### 3-(4,5-Dimethylthiazol-2-yl)-2,5-diphenyltetrazolium bromide (MTT) assay.

BCBL-1 (1 × 10^4^/100 μL) cells were seeded into 96-well plates. After treatments, the cell viability was examined by MTT assay (Sigma-Aldrich, M5655). A final concentration of 0.5 mg/mL MTT was added, and the formazan crystals were solubilized in lysis buffer (10% SDS-0.01N HCl). The optical density (OD) was determined by a microplate spectrophotometer at a wavelength of 570 and 660 nm.

### Trypan blue dye exclusion assay.

BCBL-1 cells (4 × 10^5^/300 μL) were seeded into 48-well plates. Three days after treatment, cells were resuspended by gentle pipetting and then taken out (20 μL) for cell counting. Cell suspensions were mixed with equal volume of trypan blue and counted three times by Countess 3 FL cell counter (Thermo Fisher Scientific Inc, Waltham, MA, USA).

### Flow cytometry analysis of apoptotic cells.

TREx-MH-K-Rta-shKDM4A-Flag-KDM4A-WT and -K471R BCBL-1 cells (2 × 10^6^ cells/mL) were cultured for 24 h and then treated with Dox for 48 h. To analyze apoptotic cells, 1 × 10^7^ BCBL-1 cells were washed three times with ice-cold PBS and stained with Zombie Aqua dye (Biolegend, 423109) for 15 min at room temperature. The cells were then washed three times with ice-cold PBS and fixed in 4% paraformaldehyde/PBS for 20 min at room temperature. After fixation, cells were washed once with ice-cold permeabilization buffer (Invitrogen, 88882400) and incubated with permeabilization buffer for 40 min on ice. The cells were then stained with anti-KSHV ORF 45 (Santa Cruz Biotechnology, sc53883) followed by Alex Fluor 488-conjugated AffiniPure goat antimouse IgG and with anti-KSHV ORF 8.1 (Santa Cruz Biotechnology, sc65446) followed by Cy3-conjugated AffiniPure goat antimouse IgG. The cells were then washed three times with ice-cold permeabilization buffer and analyzed by flow cytometer (CytoFLEX, Beckman Coulter) and CytExpert software (Beckman Coulter).

### Real-time reverse transcription and quantitative PCR (real-time RT-qPCR).

Total RNA was isolated by TRIzol (Invitrogen, 15596-018). cDNA was generated using the SuperScript III first-strand synthesis kit (Invitrogen, 18080-085) using Oligo-(dT). qPCR primer pairs were designed by PerlPrimer (http://perlprimer.sourceforge.net/) (CASP10-F: 5′-ATCATGTCTCACTTCACAGC-3′; CASP10-R: 5′-AGATGTCTTCATGTCTTGGGA-3′; CREB1-F: 5′-ATTAGCCCAGGTATCTATGCC-3′; CREB1-R: 5′-TGTTTGGACTTGTGGAGACTG-3′; FGFR1-F: 5′-TGCCTGAACAAGATGCTCTC-3′; FGFR1-R: 5′-ACCTTGTAGCCTCCAATTCTG-3′; GSTM1-F: 5′-GCCTGCTCCTGGAATACAC-3′; GSTM1-R: 5′-GTAGGGCAGATTGGGAAAGTC-3′; IL-10-F: 5′-CCTGCCTAACATGCTTCGAG-3′; IL-10-R: 5′-CAACCCAGGTAACCCTTAAAGTC-3′; KRT1-F: 5′-AGGGAAGGACAGTAAAGAGC-3′; KRT1-R: 5′-TTCCCATTGCTGGGATCAC-3′; NOTCH1-F: 5′-CCTCATCAACTCACACGCC-3′; NOTCH1-R: 5′-TGTCTCCTCCCTGTTGTTCTG-3′; SPHK1-F: 5′-TTATGCTGGCTATGAGCAGGT-3′; SPHK1-R: 5′-GTCTTGGAACCCACTCTTCCT-3′). The expression level of mRNA was normalized against GAPDH. Real-time qPCR analysis was carried out in the CFX96 Connect real-time PCR detection system (Bio-Rad, Hercules, CA, USA).

### Chromatin immunoprecipitation-sequencing (ChIP-Seq) and real-time qPCR.

ChIP assay was performed using protocol from Dr. Farnham’s laboratory (https://bohdan-khomtchouk.github.io/docs/Chromatin-Immunoprecipitation-ChIPs-Protocol-from-Farnham-Lab.pdf). Chromatin DNA prepared from 1 × 10^7^ TREx-MH-K-Rta-shKDM4A-Flag-KDM4A-WT and -KDM4A-K471R cells was used for ChIP assays using anti-KDM4A rabbit polyclonal antibody (25) and rabbit nonimmune serum IgG (Alpha Diagnostic International). Sequencing libraries were prepared using 10 ng of purified ChIP DNA using Illumina HiSeq Rapid PE Cluster Kit v2 (Illumina, TG-403-2001) according to the manufacturer’s instruction and analyzed by paired-end Illumina HiSeq 2000 sequencing. ChIP-Seq data were aligned to the human reference genome (GRCh38) and HHV-8 reference genome (NC_009333.1) by Partek Flow (Partek).

ChIP DNA was confirmed for successful IP using SYBR Green-based real-time qPCR analysis in the CFX Connect real-time PCR detection system (Bio-Rad). The specific primer set (5′-TTAGAGCGTTTCCAGACCT-3′ and 5′-GAGGATTCAACAGTGATGGGAC-3′) was designed to amplify the potential KDM4A binding site on the IL-10 promoter.

### IL-10 ELISA.

IL-10 was assayed in the supernatant by ELISA using a pair of antibodies (BD Biosciences, DY217B-05) following the manufacturer's instructions. The sensitivity of the ELISA was 1.5 pg/mL.
