# Supplementary material for: SUMO Modification of Histone Demethylase KDM4A in Kaposi’s Sarcoma-Associated Herpesvirus-Induced Primary Effusion Lymphoma
Source: J Virol. 2022 Aug 1;96(16):e00755-22. doi: 10.1128/jvi.00755-22 (PMC9400493; doi:10.1128/jvi.00755-22)
Supplement: Supplemental file 1 — Fig. S1. Download jvi.00755-22-s0001.pdf, PDF file, 0.1 MB [file jvi.00755-22-s0001.pdf]

Figure S1

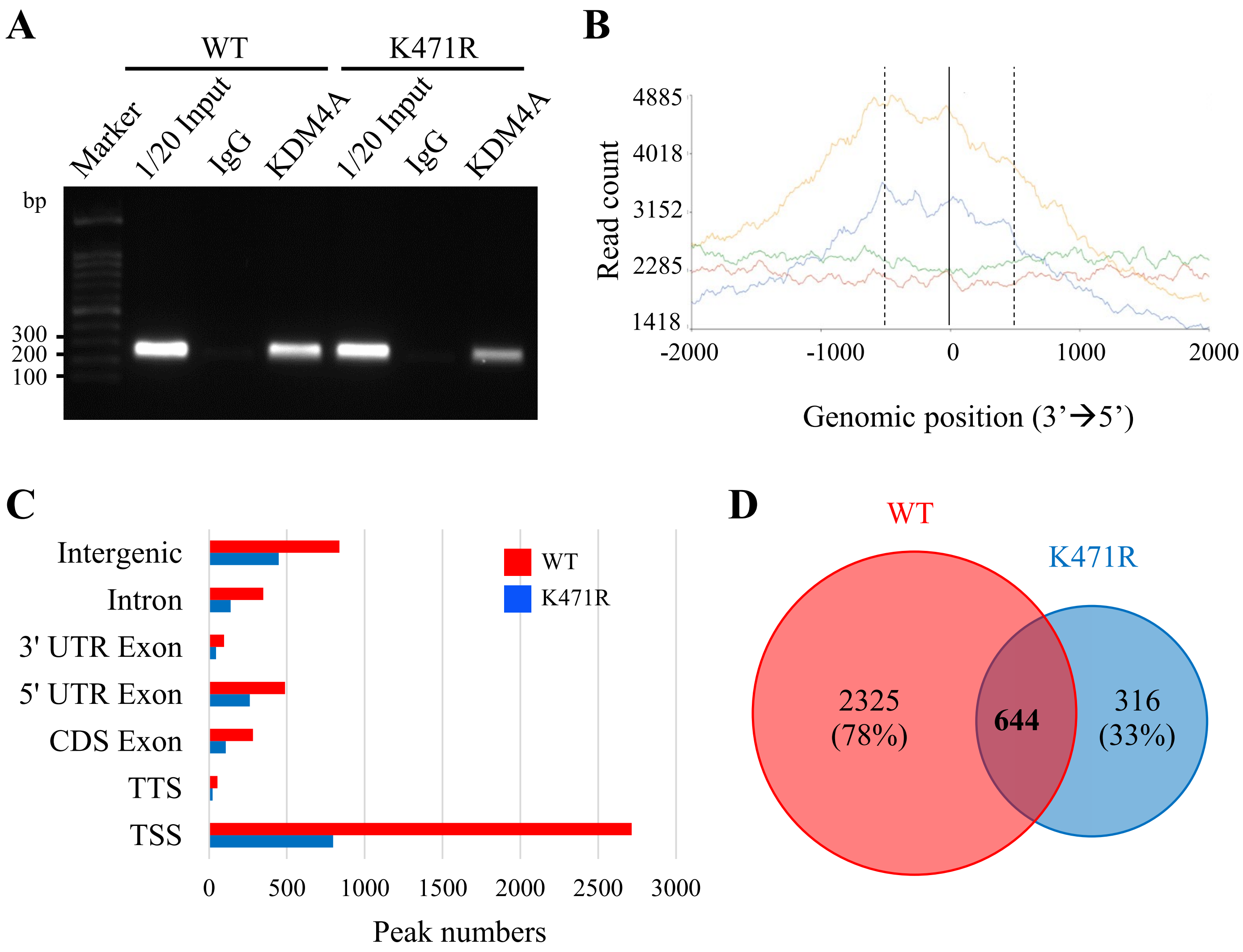

Figure S1. Genome-wide ChIP-seq analysis revealed the occupancy of KDM4A in TREx-MH-K-Rta-shKDM4A-Flag-KDM4A-WT and -KDM4A-K471R BCBL-1 cells. (A) The successful ChIP of KDM4A was confirmed by real-time qPCR using primer pairs targeting KSHV lytic gene K-bZIP. (B) Distance distribution of KDM4A peaks. (C) Gene context of KDM4A binding sites in KDM4A-WT and KDM4A-K471R BCBL-1 cells. (D) Venn diagrams depict the overlap of KDM4A binding peaks between KDM4A-WT and KDM4A-K471R.
